# High Incidence of Tuberculosis in the Absence of Isoniazid and Cotrimoxazole Preventive Therapy in Children Living with HIV in Northern Ethiopia: A Retrospective Follow-Up Study

**DOI:** 10.1371/journal.pone.0152941

**Published:** 2016-04-12

**Authors:** Yihun Mulugeta Alemu, Gashaw Andargie, Ejigu Gebeye

**Affiliations:** 1 School of Public Health, College of Medicine and Health Sciences, Bahir Dar University, Bahir Dar, Ethiopia; 2 Institute of Public Health, College of Medicine and Health Sciences, University of Gondar, Gondar, Ethiopia; University of Athens, Medical School, GREECE

## Abstract

**Objective:**

To identify the incidence of and predictors for tuberculosis in children living with HIV in Northern Ethiopia.

**Design:**

Observational, retrospective follow-up study.

**Methods:**

A total of 645 HIV-infected children were observed between September 2009 and September 2014. Cox regression analysis was used to identify predictors for developing TB.

**Results:**

The incidence rate of tuberculosis was 4.2 per 100 child-years. Incidence of tuberculosis was higher for subjects who were not on cotrimoxazole preventive therapy, were not on isoniazid preventive therapy, had delayed motor development, had a CD4 cell count below the threshold, had hemoglobin level less than 10 mg/dl and were assessed as World Health Organization (WHO) clinical stage III or IV.

**Conclusion:**

Incidence of TB in children living with HIV was high. This study reaffirmed that isoniazid preventive therapy is one of the best strategy to reduce incidence of TB in children living with HIV. All children living with HIV should be screened for TB but for children with delayed motor development, advanced WHO clinical stage, anemia or immune suppression, intensified screening is highly recommended.

## Introduction

Tuberculosis is a major cause of morbidity and mortality in people living with Human Immuno-deficiency Virus (PLWHIV) [1, 2]. In 2013, an estimated 9.0 million people in the world developed TB; 1.1 million were HIV-infected [3]. Ethiopia ranks as the seventh highest TB-burdened country in the world [4]. According to the HIV/AIDS estimates of the Central Statistical Agency of Ethiopia, a significant number of children were living with HIV in the country in 2014 [5].

Studies of the predictors for developing TB have focused mainly on the adult population [6–9]. Often in resource-limited settings estimates of TB in children have been based on extrapolation from adult data [10, 11], but children with TB vary significantly from adult TB patients in their immunological and pathophysiological responses. Pediatric TB management protocols should consider the particular epidemiology and clinical presentation of TB in children, but unfortunately epidemiological data of pediatric TB in high-burden countries are scarce [12, 13].

HIV infection increases the lifetime risk of developing TB [14]. However, HIV is not the only predictor for developing TB; various other predictors contribute to TB occurrence. Previous studies have identified sociodemographic [15, 16] and clinical [17–20] predictors for TB. Studies also have shown incidence of TB in children living with HIV [21–23]. However, among HIV-infected Ethiopian children, incidence of TB and its predictors is still not well described. Therefore, this study aims to describe incidence and identify predictors for TB in children living with HIV in northern Ethiopia.

## Methods

### Study design and setting

This is a retrospective follow-up study of children living with HIV receiving medical care in two hospitals and six health centers in northern Ethiopia between September 2009 and September 2014. Highly activated antiretroviral therapy (HAART), Cotrimoxazole Preventive Therapy (CPT), Isonizid Preventive Therapy (IPT), nutritional assessment, TB screening, CD4 cell count, and hemoglobin count were some of the clinical cares provided in the study settings. Initiation of HAART was according to the 2007 guideline for pediatric HIV/AIDS care in Ethiopia [24]. In 2015, HAART initiation criterion were revised. Subsequently all HIV positive children, regardless of CD4 cell count or WHO clinical staging, have been started on HARRT. In a monthly interval, all HIV positive children were screened for TB. The screening procedure included history taking for four targeted findings (chronic cough, documented weight loss, fever or household contact with active TB). If the child presented with any one of these symptoms, this child was a suspect TB case and was investigated according to the national guidelines for TB-HIV.

### Sampling and study participants

Out of 33 districts in West Gojjam and South Gondar Zones, five districts were randomly selected, and all health institutions in the five districts were included. Two hospitals and six health centers were identified. All HIV-infected children under the age of 15 years who were receiving medical care in the aforementioned eight health institutions were participants of the study.

### Data collection and study variables

A data collection tool was prepared in English and pre-tested for consistency and ease of understanding. Nine nurses extracted the data from patient follow-up cards and health facility log books. Four medical officers supervised the data collection process. The dependent variable was incidence of TB. Independent variables were demographic (age) and clinical (motor development, WHO clinical stage, hemoglobin level, CD4 cell count, nutritional status, isoniazid preventive therapy and cotrimoxazole preventive therapy). All independent variables were measured at baseline.

### Data analysis

Data were entered into EpiInfo 7 and analysis was done using STATA 12. Frequencies and proportions were used to describe the study subjects in relation to the study variables. The incidence rate of TB was calculated using child-years of follow-up as the denominator for the entire cohort. A Kaplan-Meier plot was used to estimate the probability of TB-free survival. To identify predictors associated with incident TB, Cox regression analysis was used. A bi-variable cox regression model was fitted for all explanatory variables. To identify confounding variables, a multi-variable cox regression model was used. All explanatory variables that were fitted in the bi-variable model were fitted to the multi-variable cox regression model. Adjustment for controlling site level variability was used by stratification (eight different health facilities). A hazard ratio with a 95% confidence interval was used to measure the strength of association and identify statistically significant results. Schoenfeld residuals were used to test for the proportional-hazard assumption (hazard is proportional if the p-value > 0.1). In this study the hazard was proportional since the p-value was 0.81.

### Case ascertainment and definitions

TB cases were identified using TB diagnostic method/s described in the Ministry of Health of Ethiopia guidelines: sputum microscopy, radiological examination or/and histopathology [24]. HIV positive children who had a follow up at least for six months in the HIV care clinic were included in the analysis. Incident TB cases were only those who were develop new TB during the follow up period. Patients who were on TB treatment at enrollment were excluded. Survival time was censored when a child dropped out, transferred, died by causes other than TB or completed follow-up without developing TB. According to the six gross motor development milestones outlined in the WHO Child Growth Standards [25], child motor development is classified into 3 categories: “delayed” if a child fails to attain the appropriate milestones for his/her age, “regressed” if a child fails to attain milestones that s/he had previously attained, or “appropriate” if otherwise. CD4 T lymphocyte was classified as below the threshold according to the following age-specific thresholds: less than 15% for children aged 12–35 months, less than 10% for children aged 36–59 months or less than 100 cells/mm3 for children aged 5–15 years [24]. A child was classified as underweight if the child’s weight for age Z score was less than -2 [26].

### Ethical considerations

Ethical approval was obtained from University of Gondar Public Health Institute ethics committee. Written permission to conduct the study was granted from each health institution involved in the study. Patient records were anonymized and de-identified prior to analysis. Patient informed consent was not required as only anonymous and operational monitoring data were collected and analyzed.

## Results

### Demographic and clinical profiles of study participants

A total of 645 patient records were included in the analysis. The median age and interquartile range (IQR) of study participants was 6 years (IQR: 3.5–9.00 years).

Table 1 presents the frequency distribution of exposure variables. A majority (606 or 94%) of children had appropriate motor development for their age. More than half (418 or 64.8%) of children were identified as WHO clinical stage I or II. A high proportion (557 or 86.4%) of subjects were on cotrimoxazole preventive therapy whereas only a small proportion (119 or 18.4%) of participants were on isoniazid preventive therapy.

**Table 1:** Baseline demographic and clinical profiles of children living with HIV in Northern Ethiopia, September 2009—September 2014 (n = 645).

Variables	Frequency (n)	Percent (%)
**Age**		
< 5 years	277	42.9
6–10 years	290	45.0
11–15 years	78	12.1
**Motor Development**		
Appropriate	606	94.0
Delayed / regressed	39	6.0
**WHO clinical stage**		
I / II	418	64.8
III / IV	227	35.2
**Hemoglobin level**		
> = 10 mg/dl	540	83,7
< 10 mg/dl	105	16,3
**CD4 cell count**		
Above the threshold	561	87.0
Below the threshold	84	13.0
**Nutritional status**		
Normal	432	67.0
Underweight	213	33.0
**Isoniazid prophylaxis**		
Yes	119	18.4
No	526	81.6
**Cotrimoxazole preventive therapy**		
Yes	557	86.4
No	88	13.6

WHO = World health organization

### Tuberculosis incidence rate and TB-free survival

**Fig 1:**
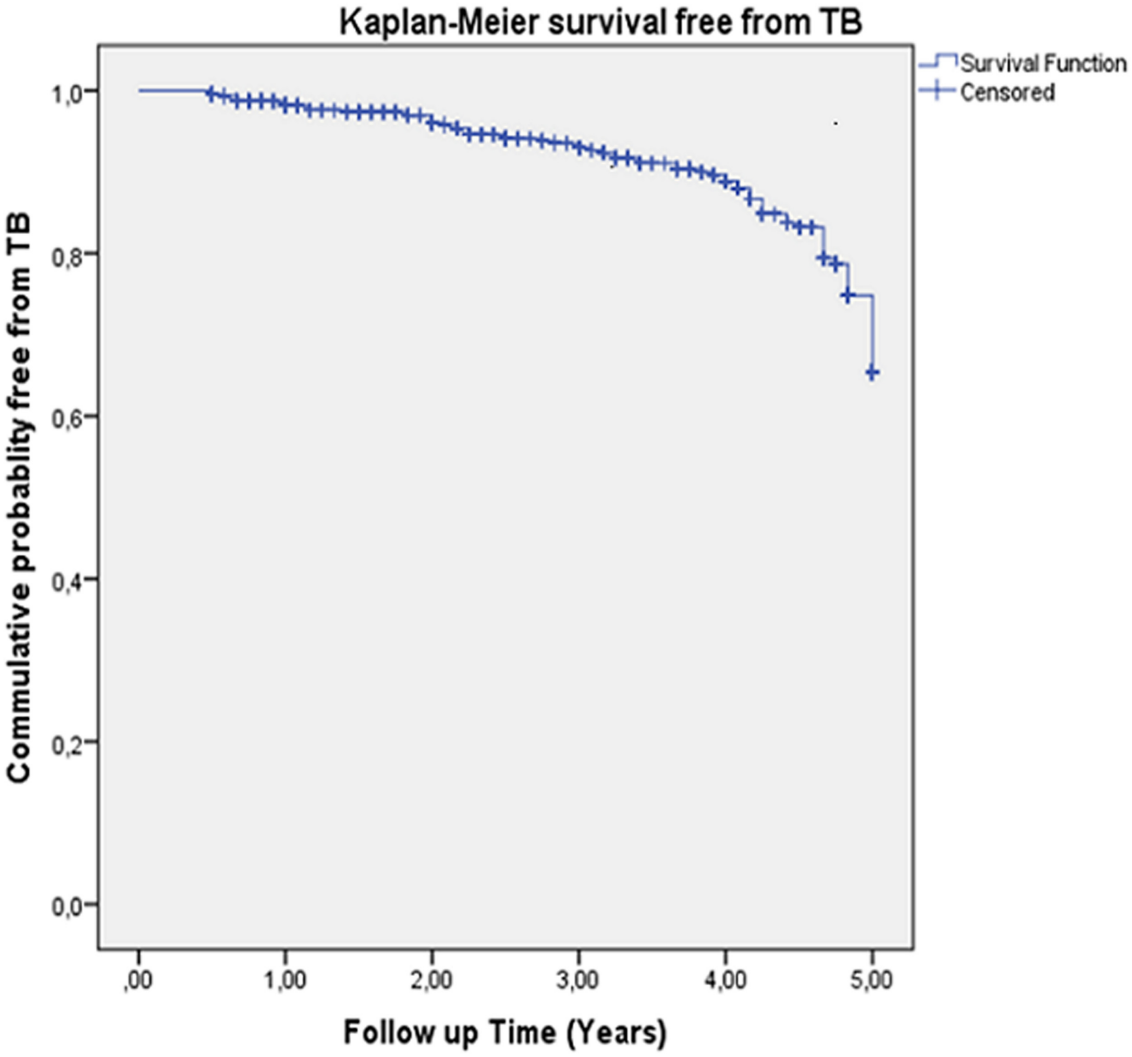
Kaplan Meier probability of TB-free survival (submitted as a separate file).

At the end of the follow up, patients lost to follow up were 6.5% (2.3% death, 2.8% transfer out, and 1.4% dropout). The median follow-up time was 36 months (ranging from 6 to 60 months, IQR = 16 to 52 months). The total child-years at risk was 1854. There were 79 new TB cases observed during follow-up. The incidence rate of TB was 4.2: 95%CI (3.4, 5.3) per 100 child-years. Fig 1 shows the Kaplan-Meier probabilities of survival free of tuberculosis: 65% by the end of follow-up.

### Predictors for tuberculosis

Table 2 shows predictors for TB. Absence of cotrimoxazole preventive therapy (AHR = 4.3; 95% CI: 2.5, 7.5) was an independent predictor for increased incidence of TB. Absence of isoniazid preventive therapy (AHR = 4.5; 95% CI: 2.1, 9.9) was also an independent predictor for increased occurrence of TB. Children with delayed motor development (AHR = 2.9; 95% CI: 1.4, 5.6) were more likely to develop TB. Patients with CD4 cell count below the threshold (AHR = 2.5; 95% CI: 1.5, 4.4) were more likely to develop TB. Hemoglobin level less than 10 mg/dl (AHR = 2.7, 95% CI: 1.6, 4.5) was linked with increased occurrence of TB. WHO clinical stage of III or IV (AHR = 2.2, 95% CI: 1.3, 3.9) was an independent predictor for increased incidence of TB. Nutritional status underweight (AHR = 1.7, 95% CI: 1.1, 2.9) was associated with increased incidence of TB. Age was not a significant predictor of TB in the multivariable analysis.

**Table 2:** Predictors for TB in children living with HIV in Northern Ethiopia, September 2009—September 2014 (n = 645).

Survival status				
Variables	Incident TB	Censored	CHR(95% CI)	AHR(95% CI)
**Age**				
< 5 years	24	253	1	1
5–10 years	45	245	0.9(0.5, 1.5)	0.8(0.4, 1.4)
11–15 years	10	68	2.5(1.2, 5.3)	1.2(0.5, 2.8)
**Motor Development**				
Appropriate	61	545	1	1
Delayed / Regressed	18	21	5.2(3.0, 8.9)	2.9(1.4, 5.6)*
**WHO clinical stage**				
I/II	25	393	1	1
III/IV	54	173	3.1(1.9, 5.0)	2.2(1.3, 3.9)*
**CD4 cell count**				
Above the threshold	47	514	1	1
Below the threshold	32	52	4.9(3.1, 7.8)	2.5(1.5, 4.4)*
**Hemoglobin level**				
< 10 mg/dl	32	73	3.5 (2.2, 5.6)	2.7(1.6, 4.5)*
> = 10 mg/dl	47	493	1	1
**Nutritional status**				
Normal	26	406	1	1
Underweight	53	160	3.1(1.9, 5.0)	1.7(1.1, 2.9)*
**IPT**				
Yes	11	108	1	1
No	68	458	2.0(1.1, 3.9)	4.5(2.1, 9.9)*
**CPT**				
Yes	45	532	1	1
No	34	34	6.8(4.3, 10.7)	4.3(2.5, 7.5)*

CHR = Crude hazard ratio

WHO = World health organization

IPT = Isoniazid preventive therapy

CPT = Cotrimoxazole preventive therapy

* Independently significant at α 0.05

## Discussion

In this study the incidence of TB in children living with HIV was 4.2, 95%CI (3.4, 5.3) per 100 child-years. The TB incidence rate observed in this study is consistent with a study conducted in Tanzania which observed an incidence of 5.2 per 100 child-years, however it is higher than the findings of a study conducted in New York City, which described an incidence of 0.61 per 100 child-years [16, 22]. This could be due to higher burden of tuberculosis in resource limited settings [4]. On the other hand, a study conducted in South Africa observed an incidence of 21.1 per 100 child years [15]. Higher prevalence of TB/HIV infection in South Africa might increases risk of mycobacterium transmission and TB occurrence [4]. A study conducted in Kenya identified an incidence of 17.5 per 100 child years [23]. In the same country but in different time period, another study conducted in Kenya, which described an incidence of 1.4 per 100 child-year [27]. Inconsistent findings might explain change of TB incidence as time goes; in some settings lower incidences of TB have been observed in recent times. Incidences of TB in HIV positive children vary from one setting to another, which ranging from 0.61 to 27 per 100 child years [15, 16, 21–23, 27–29]. Further prospective TB preventive and diagnostic studies in children are urgently needed.

Consistent with existing literature, this study identified that CD4 cell count below the threshold was an independent predictor for increased occurrence of TB [22]. Cotrimoxazole preventive therapy significantly reduces HIV related morbidity and mortality [30]. This study showed that study subjects with CPT were less likely to develop TB. Cotrimoxazole preventive therapy prevents occurrence of pneumocystis jirovecii pneumonia (PCP) [31]. In resource limited settings the rate of co-infection tuberculosis with PCP is high, which ranging from 25% to 80% [32].

We found that study subjects who were on isoniazid preventive therapy were less likely to develop TB, which is consistent with existing literatures [17, 18, 33–35]. IPT decreases mycobacterium load and reduces progression of latent bacilli to active TB [36]. Increase mycobacterial load was associated with progressive impairment of mycobacterium specific T cell response and increased occurrence of active TB [37]. In settings where latent TB infection is not adequately treated, children infected with TB will provide reservoir for future TB cases [38]. In a situation where prevalence of latent TB infection is high, guideline for pediatric HIV/AIDS care and treatment in Ethiopia recommends IPT for HIV positive children in whom active TB has been excluded [24]. In this study only 18.4% of the HIV positive children received IPT. This study therefore highlights the need to increase the uptake of IPT.

Patients at WHO clinical sage III or IV were more likely to develop TB compared with children at WHO clinical stage I or II. Patients with CD4 lymphocyte count was less than 350 cells/ml were more likely assessed as WHO clinical stage III and IV [39]. Advanced HIV disease is linked with immunological deterioration [40, 41].

In this study underweight was a predictor for increased occurrence of TB. This is consistent with studies in Cote d'Ivoire, Tanzania and Kenya [16, 21, 23]. Nutritional status was associated with CD4 T cells recovery [42]. TB is an opportunistic infection; incidence of TB is higher in patients with immunosuppression [22]. Malnutrition decreases the likelihood of adequate motor development in HIV-exposed children [43]. Furthermore, among HIV positive children, weight-for-age Z-score was a stronger predictor of neurocognitive outcomes [44]. This study identified that delayed motor development was an independent predictor for increased incidence of TB. It is consistent with a study done in Indian; developmental delay was associated with increased occurrence of TB [19]. As a child becomes adolescence or adult, under-nutrition and poor developmental milestone have been associated with adverse economic impact due to decreased human productivity and low wages [45]. In resource limited setting, tuberculosis is more common in the lower-income group [46].

Previous studies conducted in Tanzania and South Africa identified that children diagnosed with anemia were more likely to develop TB [16, 20]. Similarly our study showed that hemoglobin level less than 10 mg/dl was an independent predictor for increased incidence of TB. Severe anemia was associated with poor clinical outcomes [47]. Among people live with HIV/AIDS, low level of CD4 count was a predictor for anemia [48]. HIV infection in children is often complicated by anemia, which can result from the direct effect of HIV on bone marrow cells, HIV-related opportunistic infections, or certain antiretroviral drugs. The negative impact of HIV on anemic children worsen with duration of HIV infection; the risk of anemia-associated morbidity and mortality may increase when the child become adolescent or adulthood [49–51]. The mechanisms for TB associated with anemia need to be evaluated through further research.

This study has the following potential limitation: as this was an operational research study and children were followed in routine medical care; entry into follow-up was not triggered by common clinical criterion. Therefore the sample is not homogenous in relation to several factors and there may be some bias in analysis of predictors.

## Conclusion

Incidence of TB in children living with HIV was high. This study reaffirmed that isoniazid preventive therapy is one of the best strategy to reduce incidence of TB in children living with HIV. All children living with HIV should be screened for TB but for children with delayed motor development, advanced WHO clinical stage, anemia or immune suppression, intensified screening is highly recommended.

## Supporting Information

S1 Text Data of incidence of TB in HIV positive children (XLSX)
